# The Intellectual Prerequisites for the Student of Medicine

**Published:** 1885-11

**Authors:** Thomas Lothrop

**Affiliations:** Professor of Obstetrics


VOL. XXVI. NOVEMBER, 1885. No. 4.

### Original Communications.

### THE INTELLECTUAL AND MORAL PREREQUISITES FOR THE STUDENT OF MEDICINE.

An Introductory Lecture, delivered at the Medical Department of Niagara University, at the opening of the Medical Session of 1885-86.

BY THOMAS LOTHROP, M. D., Professor of Obstetrics.

*Gentlemen of the Medical Class:*

The Medical Faculty have conferred an undesired and unmerited honor in selecting the speaker to deliver the introductory lecture at this, the opening of the Third Annual Session of Medical Lectures. It affords me a much-coveted opportunity to look over a new class, to estimate the quality, as well as the quantity, of the material of which it is composed, and to look squarely into the faces of those to whom it will be my privilege, during the present session, to unfold the principles of an important coördinate department of medical science.

It is, indeed, a pleasure to be ushered into the presence of a throng of young men, such as I see before me, gathered from widely distant localities, to engage in a course of study of such profound interest as that of medicine; and the inquiry is naturally suggested on such an occasion, and its expression becomes almost spontaneous, what motive has influenced you to turn your steps towards this temple of science ?

This inquiry brings up a fundamental question, from which every motive which has entered into the formation of your resolution spring.

What principle or motive turned your attention towards the study of medicine ? I will not presume to enter into an analysis of the circumstances which have led each of those, to whom it is my privilege to speak this evening, to the selection of medicine for a study and for a life-work. May I hope that, in this examination, there will be found in the depths of your hearts, as the controlling impulse, a profound love of science, a fondness for scientific research, and a deep reverence for nature, as exemplified in the organization of man ; and that your minds will be broad and comprehensive enough to take in the causes, intrinsic and extrinsic, which produce morbid phenomena.

But there is another inquiry, quite as pertinent as the first I presented to you: Why do you come here ? It is almost, at this juncture, too simple to mention or to repeat. It has now become a part of the accepted alphabet of your existence. Men need to be educated in the science of medicine, because, being ignorant of themselves, of their marvelous physical organization, of disease, and of the causes leading thereto, they need the discipline we are prepared to give and the instruction we are selected to impart. You seek to be educated in medicine because of your ignorance: of your ignorance of yourselves ; of your physical being and the laws of your development and growth, and, also, of your environment, which leads to health with its blessings, or to disease with its manifold sufferings. To fulfill the grand object of a life, consecrated to a science as broad and comprehensive as that of medicine, you need to know intimately and thoroughly—first, its fundamental principles, and, second, the application of those principles to the varied conditions to which the human race are subjected.

To do this work rationally and wisely, you require positive knowledge; and, being confessedly ignorant, you need to learn; and, being ignorant and having to learn, it follows that you must be educated, trained and disciplined. It may be also asked, in this connection, what do you need to know? and I answer just that of which, on entering the portals of the profession, you are supposed to be ignorant, namely: Yourselves, the physical structure and organization with which the Creator has endowed you, the laws of your being, and those widespread deviations from a normal standard which we term disease.

A true medical education first includes a thorough knowledge of man in all the structures and functions with which he is endowed; and, secondly, of man under those perverted conditions which we comprehend under the term disease.

How is all this work, so wide in its scope and comprehensive in its purpose, to be accomplished?

First—Through didactic lectures and recitations.

Second—Through clinical instruction and observation.

This course of instruction presupposes the existence of at least one fundamental principle or fact, that the mind of the student has previously received a thorough training, or, in other words, the mental discipline which results from an academic or collegiate education, by means of which the mind is able to know its own powers, and to use those powers to their full capacity. Such a discipline not only favors the acquisition of knowledge in your chosen profession, but expands and broadens you so that you may be an earnest student in the collateral sciences.

Following, as a consequence of this possession of a liberal education on the part of the student, is the logically clear judgment, the habit of industry, the full command of your abilities when required by the exactions of difficult problems in medical science, the delicate, refined tastes, the genuine tact; in fine, those ripened results of intellectual culture which demonstrate that you have the capacity to act as free, well-balanced, rational men, capable of forming a logical opinion and of deciding the manifold questions arising in the special field of knowledge in which you are engaged.

The prevalence of popular errors or isms in medicine, now so widespread over the land, and the crude and fanciful notions existing in almost every community in regard to disease and remedies, may be traced primarily to the fact, now generally acknowledged by the profession, that half-educated men have been turned loose from our medical colleges who were unable to trace effects back to causes, and were also incapable of tracing a rational pathology in even ordinary diseases, as observed in their daily experience. One cannot wonder that such minds conjure up hidden and irrational causes to shed light upon strange and unusual manifestations of disease; make a plausible guess, which to them is equivalent to positive proof, while one happy hit, well advertised, counterbalances a thousand mistakes and failures.

Clinical instruction, the study of disease at the bedside or in the amphitheatre, furnishes the practical knowledge and useful experience which can be applied in the varied exigencies of professional life. Success here depends upon the quickness of discernment and perception, soundness of judgment in differentiating various morbid conditions, while the readiness of application of remedial agencies completes the solution of the problem.

*Clinical medicine* holds, with this college, an important position in the education of medical men for their special work. It points out the manner of obtaining evidences of disease, and provides, by actual examples, the methods of recognizing different morbid conditions, and explains the indications for treatment. Clinical medicine, therefore, comprises diagnosis and special therapeutics.

But of primary importance to you who are entering now upon the study of medicine, is the *mental discipline which gives the power to acquire further knowledge.*

" The supposed acquisitions, observations and reasonings of a man with an untrained mind, are necessarily crude and imperfect, and are often useless."

The unskilled and untrained hand is as clumsy in its manipulations of the scalpel, the bandage, or the splint, as the untrained mind is in analyzing the problems which are constantly presented in practice. I confidently assert that the student who is possessed of a fair intellectual culture will, in time, possess that maturity of judgment which will enable him to use his powers wisely, calmly and advantageously.

These principles are fundamental. The recognition of them inspired the establishment of the department of medicine in Niagara University. The motto "Higher education" is no figment of fancy with its faculty, but the firm, unalterable conviction that both professional and public sentiment call for its rigid enforcement.

The trustees and faculty recognize that mental discipline, prior to entering upon the study of any of the learned professions, is *the primary condition* of admission into their ranks. We, as a faculty, are directly responsible for the results which flow from your continuing the study of medicine under our guidance. We accept that responsibility with a full realization of the consequences it entails to the profession and to the public.

In the political world, with all the selfishness and chicanery which have dragged the body politic down to its present low level, there is observed the rising out of the ruins a principle which aims to make fitness and capacity and honesty the conditions of public service. The perpetuity of our institutions demand the reform. The safety of the republic call for radical changes. "Civil Service Reform" is the motto of the better elements of the political parties of the day. The need of it is recognized and candidly acknowledged by statesmen and by the people, and within a few years, even selfish politicians have been compelled to adopt the principle. Comparing the present condition of the medical profession of this country, and the mass of crude material which the medical colleges, freed from State control, have poured out upon the community, with the political world, the intelligent observer can recognize the need of the adoption of like measures for the accomplishment of like results. Higher education leads to the selection of better material to fill the rank and file of the profession. *Better material* means *better work*, and the profession then will rise to the high position in the estimation of the public to which its noble purposes and aims entitle it.

I have emphasized, thus far, in my address one principle to be essential to raise the standard of professional excellence in medicine. Intellectual qualifications would appear the sine qua non for our science. You may well put the inquiry, "Is there nothing more that is essential in a profession so humane in its purposes and so noble in its principles, than the cultivated intellect, the mature judgment, the ripe knowledge, which comes from careful study and observation?" These qualities, if I may so term them, have been known to drag the degree of doctor of medicine from its high estate down to the scaffold—there to expiate for crimes, the commission of which professional erudition materially assisted. The prison and the dungeon are to-day occupied by men of marvelous medical genius. The crafts and subtleties of the professional charlatan, quickened by intellectual power and force, find in every community willing victims and profitable pecuniary returns. The reign of imposters, with greed for their motto, even though thousands of precious lives are sacrificed as victims to unscrupulous empiricism, flourishes at the present day as never before in the history of these latter centuries.

Well may we stop for a moment, to reflect upon the causes leading to a state of things as painful to the philanthropist as it is humiliating to the profession. History repeats itself in the lives of individuals, as in the growth and decay, the rise and fall, of nations.

Ancient Greece furnished the intellectual accomplishments which made Athens the pride and glory of the world, and the prowess of Rome, in the family of nations, received its inspiration from the marvelous strength and intellectual force of its statesmen, its men of letters, and its soldiers.

Something more than intellectual culture, then, is wanted to raise the science and art of medicine to the high position which it should command, by virtue of its grand and beneficent purposes and objects. Among its votaries should be found the manliest of the race, even as the benefits bestowed through its instrumentality upon the individual and upon the community are the outcome of the noblest and best of human efforts.

Medicine, through the blessings it diffuses far and wide over the earth, calls forth the highest and noblest elements of human character. It is, indeed, a manly art, and its history, traced from the earliest period of Greek civilization, in which the Homeric heroes are represented to have possessed considerable knowledge of surgery, antedating the Hippocratic period, down along the course of subsequent centuries, have, at all times, found the art an important factor in the civilization of the race.

The history of medicine, from the dawn of creation to the present time, is also coincident with the history of human suffering. As the human race have groaned and travailed in pain, the human mind has been stimulated to effort for its alleviation. The purpose of disease is the divine economy; is one of those mysteries which the mind cannot comprehend and will never unravel.

But that pain and suffering have subserved some great end, none but the fool would dare to deny. The influence of suffering brings out human sympathy and joins men in closer relationship, the one with the other. It breeds gentleness, it inculcates purity, it teaches the utter helplessness of the subject, it cleanses and purifies. In such an atmosphere, the medical man mingles, breathing the blessed influences of this divine mystery. Out of pain and anguish and torture, man rises, chastened with the divine hand, and the better prepared to fill the niche for which he was created. Upon this foundation, medicine has risen, and the superstructure reared thereon possesses the fair proportions, marred here and there by occasional defects, of the temple of science. The Manger and the Cross gave the sanction of omnipotence to the mystery of suffering, while, at the dawn of the first Easter morn, there was traced on the horizon, with the divine finger, to be seen of men, the solution of that problem which makes snffering the antecedent of joy — an epoch in the world's history, from which has grown the latest and best, because it is the civilization of man under the special inspiration of Heaven.

I have said that medicine is a manly art, calling for the exercise of the highest and noblest virtues of its votaries. You must recognize at once why it is so. While not presuming to solve the mystery of suffering, it strives to mitigate and soften its effects upon mankind. It aims to apply the balm to heal the wound; it labors to make human life happier by ministering to the diseased and by prolonging life; it enters into the sacred precincts of family and domestic life, and assuages sorrow, alleviates pain, and thwarts the fatal tide of disease; it fosters and protects the products of blessed union, and wards off the darts of the destroyer, that length of days may be given and healthy growth supplement the arrested march of disease.

Only the best of that which makes the complete man, the clearest intellect, thoroughly trained and disciplined, the moral nature, guided by a recognition of man's responsibility to God's law, the physical stature, grown to the most symmetrical of manly proportions, should become devotees at such a shrine. How far the best fall short of such an ideal, I will not presume to say. How much higher in the scale of moral and intellectual greatness and excellence we should aim, the written Word only will direct.

To the fundamental principle to which I first directed attention, as a prerequisite for the matriculant in entering upon the study of medicine, the trained and disciplined mind, is, therefore, that second factor, the *educated conscience*, which guides the moral forces wisely through the labyrinths of the temple of medical science. The profession needs not only a higher order of intellectual attainments, but it demands, in order that it may fill its mission, the moral forces quickened, so that the law of nature will be, at all times, recognized to be the law of God.

The mind and the heart are twin forces in the complex mechanism of man, and not often of symmetrical development. Intellectual genius and moral obliquity are often associated. The one may be unduly developed, while the other dwindles and decays. Such monstrosities are found everywhere. The profession may and should recognize the beautiful symmetry portrayed in the life and character of the ONE PERFECT MAN. It is the divine model for imitation. He who patterns after such an ideal reaches nearer the type of intellectual and moral greatness which medical science demands for its votaries.

Such is the standard of true manliness I have the right to ask you to imitate. When strength was given to the palsied limb and sight to the blind, the injunction followed "that they should tell no man"—an ethical law springing from a fountain of divine equity and justice, which should command recognition and obedience by all who accept the inspiration of God's Word.

Medicine, as a manly art, follows from the adoption of the principles I have thus endeavored to enunciate, and, as an active and powerful instrumentality in the alleviation of distress and the amelioration of the condition of mankind, she becomes at once the hand-maid of religion. Its special work, "to heal the sick," calls for the highest and manliest of human virtues, the best material, the noblest of human attributes, if it fulfills its mission; and I firmly believe that in its ranks are found conspicuous examples of the noblest heroism, as well as of earnest effort, to imitate in daily life the Good Physician whose life was a loving and beautiful exemplification of heavenly virtue.

Let me stop here, in conclusion, and ask the question, Is the standard of intellectual and moral excellence I have presented, too high and beyond the reach of human attainment? Are my aims for medicine as an *art* the dreams of an enthusiast? Is the ideal I offer the work of fancy or the product of a morbid imagination?

In these latter days, when the world is prone to materialism, and mankind is absorbed in present and selfish needs, and carried away with the enterprise of commerce or the discoveries of science, is it strange that medicine should catch the inspiration of the times and be turned into forbidden paths?

Heaven, in its mysterious guidance of human efforts, may point the finger, while man, individually and collectively, may fail to comprehend the meaning thereof. The whole tendency of scientific work and labor portrays a failure to acknowledge responsibility to a higher source. Man fails to watch the compass which points towards the goal, whence flows the hidden truths of nature.

A word in regard to our college and its work, and I have done. We commence to-night another year of labor in the cause of science and humanity, under auspices the most flattering, and with encouragement beyond our most sanguine expectations. Our work here was inaugurated with the single motive, the elevation of the profession, and that end has been constantly held in view. The profession and the public have given ample proof of their approval, which has stimulated us to greater effort to achieve success. We have met with obstacles which would dismay any but the most resolute. What great work for science and humanity has failed to receive assaults from selfish and designing men? We have been maligned, only to find that hindrances placed in our way, the darts hurled at our work, and the malignant statements of dishonest men, have given publicity to our principle and added prosperity to our labors.

Supported by a strong and growing sentiment in the profession, and aided by the prayers of holy men, with whose paternal blessings this department has been launched on its mission of mercy, we will strive, until success, based on righteous principles, is attained, and the profession and the public more widely acknowledge our merits. We aim only for success, which comes from the highest principles of action. We ask no recognition, unless it is deserved. We send no person forth from our halls bearing the insignia of the medical profession, unless fully equipped for its responsible duties, and we hope that those whom we honor with our recognition will go forth to their work, to heal the sick,—a mission which the angels may well smile upon,—actuated by the highest principles and possessed of manly virtues, which will receive the approval of God and man. Only by the faithful performance of the responsible trusts which are reposed in us as teachers, can we expect returns, which will commend us to you as students, and to the good will of the profession and of the public as the faithful custodians of sacred interests.

